# Recent advances in the biomedical applications of black phosphorus quantum dots

**DOI:** 10.1039/d0na01003k

**Published:** 2021-01-26

**Authors:** Yuhang Miao, Xiaojing Wang, Jie Sun, Zhong Yan

**Affiliations:** Institute of Materia Medica, Shandong First Medical University, Shandong Academy of Medical Sciences Jinan 250000 Shandong P. R. China sunjie310@126.com; College of Materials Science and Engineering, Nanjing University of Science and Technology Nanjing 210094 China zhongyan@njust.edu.cn

## Abstract

Zero-dimensional (0D) black phosphorus quantum dots (BPQDs), the new derivatives of black phosphorus (BP) nanomaterials, have attracted considerable attention since they were first prepared in 2015. Compared to traditional two-dimensional (2D) BP nanosheets, BPQDs exhibit some unique properties and demonstrate great potential for a broad range of applications, especially in the field of biomedicine. Due to the rapid development and substantial research interest in this area, it is urgent to review the current advances, challenges and near-future possibilities of BPQD-related biomedical research, which will benefit the further development of this field. This review is mainly focused on the latest progress of BPQD related applications in the biomedical field, including photothermal therapy (PTT), photodynamic therapy (PDT), drug delivery, biological imaging, *etc.* The challenges and future prospects are also discussed.

## Abstract 2

Zero-dimensional (0D) black phosphorus quantum dots (BPQDs), the new derivatives of black phosphorus (BP) nanomaterials, have attracted considerable attention since they were first prepared in 2015.

## Graphical abstract

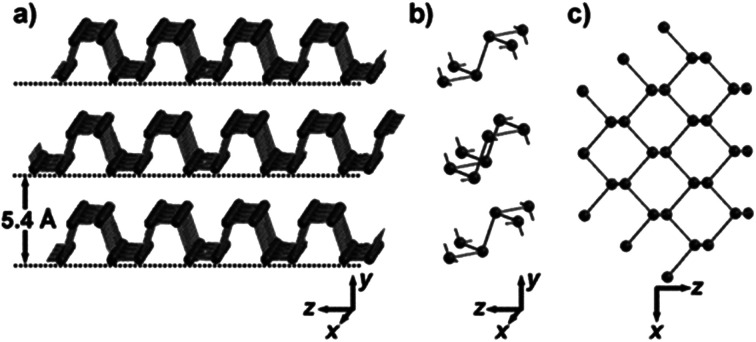

## 1. Introduction

**Fig. 1:**
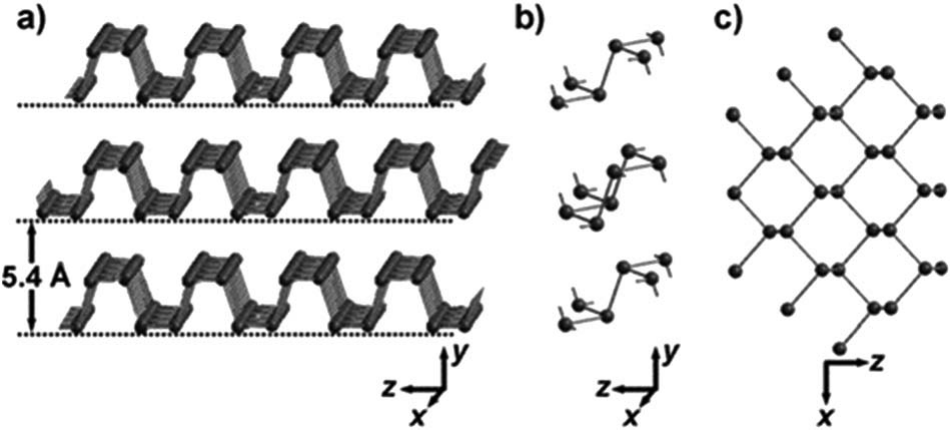
The layered crystal structure of black phosphorus (BP). (a) BP crystal. (b) Three adjacent folded plates with connected phosphorus atoms. (c) Top view of (a).^1^ (Copyright 2015, *Angew. Chem. Int. Ed.*).

Bulk black phosphorus (BP) was first synthesized in 1914. It is the most stable allotrope of phosphorus compared with white and red phosphorus. Bulk BP is a crystal material with a layered atomic structure composed of phosphorus atoms, in which individual atomic layers are bonded through weak van der Waals interactions, while in each layer, phosphorus atoms are covalently bonded to form a puckered honeycomb structure (Fig. 1). A two-dimensional (2D) BP material was first reported in 2014 and attracted enormous attention owing to its extraordinary electronic properties. BP is a direct band gap semiconductor with thickness dependent band gaps, varying from 0.3 eV (monatomic layer) to 2 eV (bulk) with a number of atomic layers. A direct band gap means that electrons in BP can directly interact with photons without the participation of phonons to compensate for momentum mismatch, so high-performance light-emitting devices can be fabricated.^1–3^ Moreover, semiconducting BP with high carrier mobility has a wide application prospect in thin film transistor devices.^4–6^ In recent years, with the in-depth studies of 2D BP materials, researchers have also discovered many other special properties of BP, such as anisotropic mechanical and electrical properties,^2^ negative Poisson's ratio,^7^ and excellent nonlinear optical properties,^8–11^ among others. Its applications have been extended to solar cells,^12^ lithium/nano-ion batteries,^13–15^ electrochemistry, gas detection,^16,17^ stress sensing, ultrashort pulse laser generation and some other fields.^18,19^

In addition, the application of BP in biomedicine has also been intensively investigated. BP is a nanoelemental material composed of phosphorus, a very important element in the human body. Phosphorus is an important component of nucleic acid and a key genetic material. Phosphorus is also an important component of adenosine triphosphate (ATP), the direct energy source of various life activities.^20^ Meanwhile, phospholipid is the main component of biofilms and phosphorus can also be found in enzymes, bones and teeth.^21,22^ In summary, phosphorus is one of the indispensable substances that constitute the human body and maintain its life activities.^23,24^ Therefore, in principle, BP should have better biocompatibilities compared with other 2D nanomaterials. In recent years, owing to its remarkable properties such as a layer-dependent bandgap, large surface-area-to-volume ratio,^25–27^ biodegradability, intrinsic photoacoustic properties,^28–31^ and biocompatibility,^32–34^ BP has attracted enormous attention for various biomedical applications including photothermal therapy (PTT),^35–39^ photodynamic therapy (PDT),^40^ drug delivery,^41–45^ bioimaging, biosensing,^46^ and combined immunotherapy, among others.^47^

**Fig. 2:**
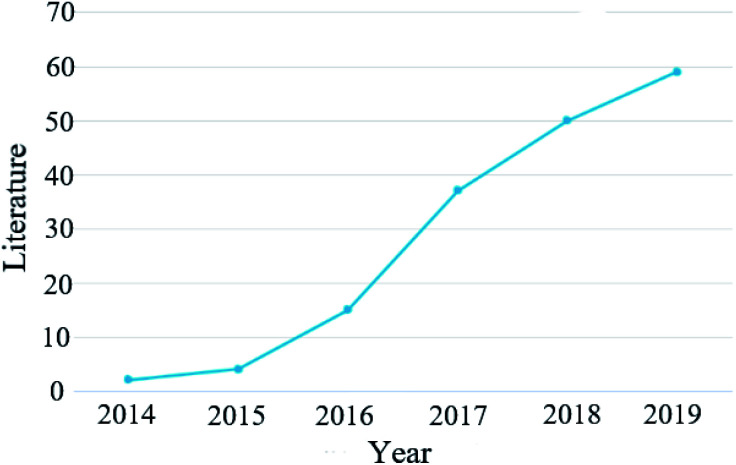
The number and classification of articles related to BPQDs since 2014.

Inspired by the successful preparation of 2D BP, zero-dimensional (0D) BP quantum dots (BPQDs), another nanostructure of BP, were first synthesized by Zhang and co-workers in 2015 through a liquid-phase ultrasonic technique.^1^ After that, Sun *et al.* discovered the efficient photothermal conversion behavior of BPQDs in the near infrared region (NIR), indicating that BPQDs have great potential for application in photothermal cancer therapy.^48^ Since then, BPQDs have been widely studied in various fields and the number of articles published has increased rapidly (Fig. 2). In recent years, two-dimensional black phosphorus nanosheets have also shown important biomedical significance. Black phosphorus nanosheets are easy to functionalize and have a large specific surface area, which can adsorb chemotherapeutic drugs and have good photothermal properties, so they can be used in photothermal therapy of tumours. Controlling near-infrared light can regulate black phosphorus nanosheets to cross the blood–brain barrier to achieve intracerebral therapy. BPQDs have excellent near infrared optical properties. Under the irradiation of a near-infrared laser, they can significantly kill tumour cells. They show good biocompatibility in a variety of cell lines.^49–52^ Compared with black phosphorus nanosheets, BPQDs have a higher specific surface area and more surface-active sites, which is beneficial to improve the efficiency of photocatalysis, and have higher photocatalytic flexibility and more favourable surface modification and functionalization. BPQDs have exhibited many extraordinary properties and demonstrated great potential for multidisciplinary biomedical applications. Although black phosphorus nanomaterials have shown great potential in biomedical applications, they still face challenges in terms of biosafety and environmental stability. Because of the rapid development and huge research interest in this area, it is urgent to review the current advances, challenges and near-future prospects of BPQDs, which will benefit the further development of BPQD-related biomedical research. In 2018, Gui *et al.* discussed the synthesis, functionalized modification and applications of BPQDs in a review article.^89^ In 2019, Luo *et al.* published another review paper focused on the biomedical applications of 2D BP, in which some results related to BPQD biomedical studies were also mentioned.^53^ The current review is mainly focused on the latest progress of BPQD related applications in the biomedical field, especially those reported in the last two years. The challenges and future prospects are also discussed.

## 2. Strategies to improve the stability of BP

**Fig. 3:**
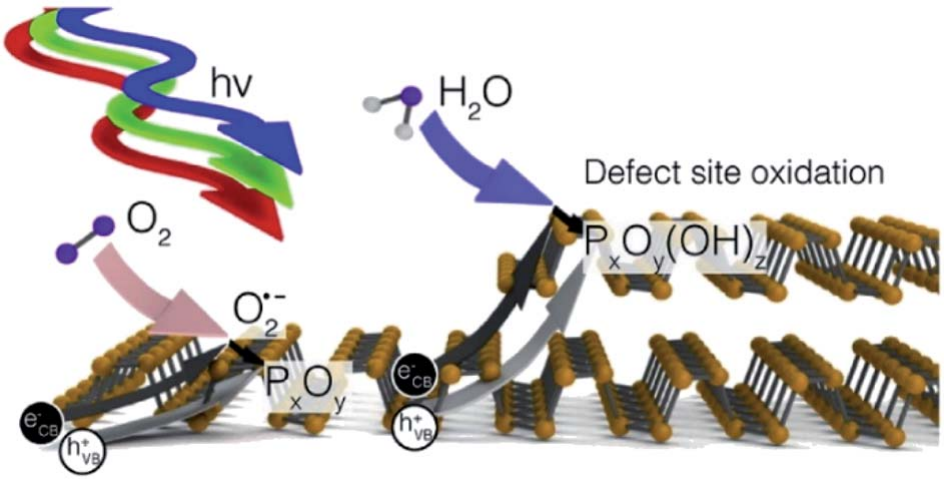
Degradation process of black phosphorus.^55^ (Copyright 2017 *J. Am. Chem. Soc.*).

The stability of BP is closely related to the application of BPQDs in biomedicine. Although BP has great application potential, there still exist bottlenecks hindering the practical application of BP. Among them, the instability in air and water environments and in human circulation has become the focus in the biomedical applications of BP and BPQDs.^54^ As shown in Fig. 3, due to the folds of the honeycomb structure, one phosphorus atom of BP is covalently bonded to three other monolayer phosphorus atoms, but the exposed lone pair electrons can react with oxygen to form P_*x*_O_*y*_ and P_*x*_O_*y*_ and water to form a phosphate, resulting in BP degradation.^55^ At present, the main stable ways to maintain the inherent characteristics of BP include the formation of hybrid systems with Al_2_O_3_, TiO_2_, sulfonated titanium ligands (TiL_4_), polyimides or aryl diazo functional groups.^56^

**Fig. 4:**
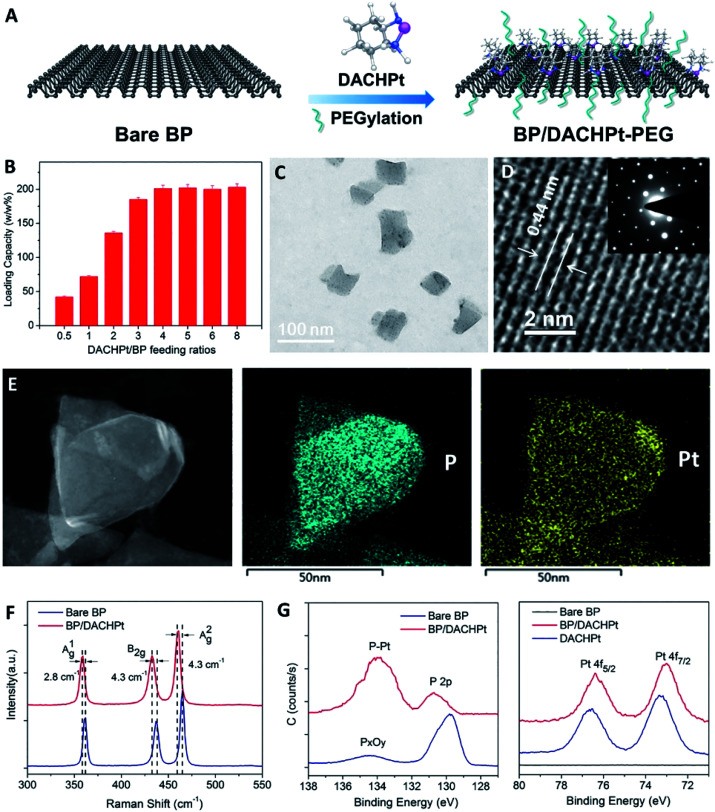
Fabrication and characterization of BP/DACHPt. (A) Surface coordination of DACHPt and PEGylation on BP. (B) DACHPt loading capacities on BP nanosheets (w/w%) with increasing DACHPt/BP feeding ratios. (C) TEM image, (D) HR-TEM image with inset SAED patterns, and (E) STEM and EDS mapping (scale bar = 50 nm) of BP/DACHPt. (F) Raman spectra of bare BP and BP/DACHPt. (G) HR-XPS spectra of P 2p and Pt 4f.^57^ (Copyright 2019, *Chem. Eng. J.*).

In recent years, the strategy of building a stable BP system has been continuously improved. Liu *et al.* used the active species of platinum anticancer drugs (DACHPt and Pt(NH_3_)_2_) to form a BP complex with BP nanoparticles. Through the novel strategy of stabilizing BP of the drug itself, the potential risk of clinical application can be avoided, and a stable BP-based drug delivery system can be constructed. The experimental results show that the complex plays a role in tumour ablation *in vivo* (Fig. 4).^57^

Although we have some strategies to improve the stability of BP, there are still doubts about the nature of BP stability, which is also an important direction of BP research in the future. Therefore, it is necessary to further understand the safety, long-term toxicity and biocompatibility of BP.

## 3. Biomedical applications of BPQDs

### 3.1 Photothermal therapy (PTT)

Photothermal therapy is a therapeutic method that uses targeted technology to enrich high photothermal conversion materials in tumor sites and converts light energy into thermal energy under the irradiation of an external light source, which leads to thermal ablation of cancer cells.^58–60^ PTT has attracted wide attention in the past few years because of its few side effects and high efficacy and has become a promising alternative to traditional cancer therapy.^61–65^ Right after the experimental preparation of BPQDs, Sun *et al.* discovered that BPQDs had a broad absorption band that spans the UV to NIR region. Ultra-small BPQDs can rapidly convert NIR light into thermal energy and have excellent NIR photothermal performance.

**Fig. 5:**
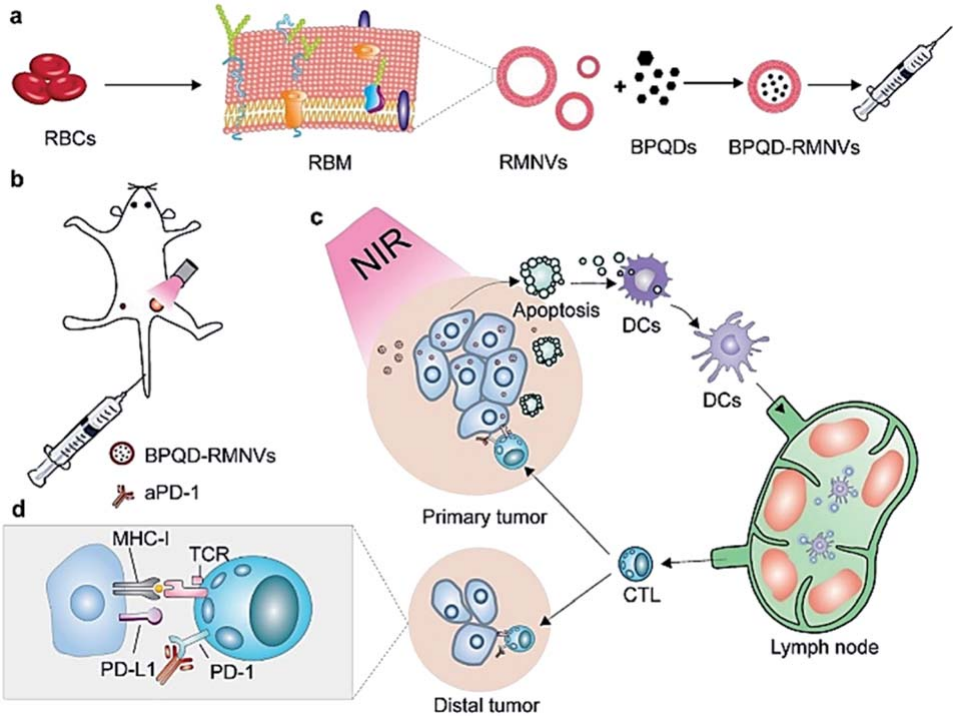
Schematic diagram of photothermal cancer immunotherapy mediated by BPQD-RMNV and aPD-1. (a) Preparation of BPQD-RMNV. (b) 4T1 tumour-bearing mice treated with BPQD-RMNV-mediated PTT and aPD-1. (c) PTT induces tumour cell apoptosis and antigen release *in situ*; DCs are recruited to present the antigen to natural T cells in lymph nodes. (d) APD-1 protects tumour infiltrating CD8 + T cells from the depletion of the tumour ligand PD-L1.^66^ (Copyright 2019, *J. Controlled Release*).

**Fig. 6:**
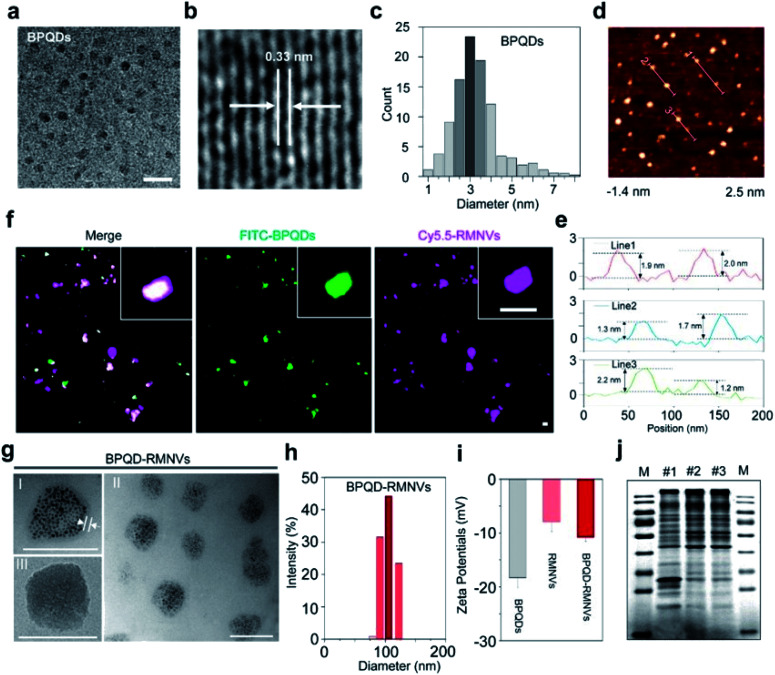
Characterization of BPQD-RMNVs for PTT. (a) TEM (scale bar, 20 nm) and (b) high-resolution TEM images of a BPQD (scale bar, 10 nm). (c) The size distribution of the BPQDs based on the TEM images. (d) AFM image and height profiles of the BPQDs (scale bar, 200 nm). (e) Zeta potential of the BPQDs. (f) Confocal image indicating that the BPQDs were coated with RMs. Cy5.5 and FITC were used to label RMs and BPQDs, respectively (scale bar, 100 nm). (g) TEM image of the BPQD-RMNVs; the inset displays the BPQDs (scale bar, 100 nm). (h) Size distribution of BPQD-RMNVs as measured by DLS. (i) Zeta potential of BPQD-RMNVs. (j) Proteins in empty RBCs (I), RMNVs (II) and BPQD-RMNVs (III) resolved on a polyacrylamide gel. M: Protein marker.^66^ (Copyright 2019, *J. Controlled Release*).

Combined with excellent biocompatibility, BPQDs were ideal thermal agents in PTT applications. Since then, BPQD based PTT study has developed very rapidly and still remains the most active research topic of BPQD related biomedical applications. Many new achievements in this field have been reported in the last two years. Liang *et al.* prepared a BPQD-RM (erythrocyte membranes, RM) nano-vesicle (BPQD-RMNV) biomimetic formulation, which was irradiated with a near-infrared (NIR) laser to induce the apoptosis of breast cancer cells *in situ*, through mobilizing the immune system to eliminate residual and metastatic cancer cells. A programmed cell death protein 1 (PD-1) antibody (aPD-1) was used to prevent the depletion of CD8 + T cells (Fig. 5). The results showed that BPQD-RMNV-mediated PTT combined with aPD-1 therapy could significantly delay the growth of residual and metastatic tumors *in vivo* (Fig. 6).^66^

**Fig. 7:**
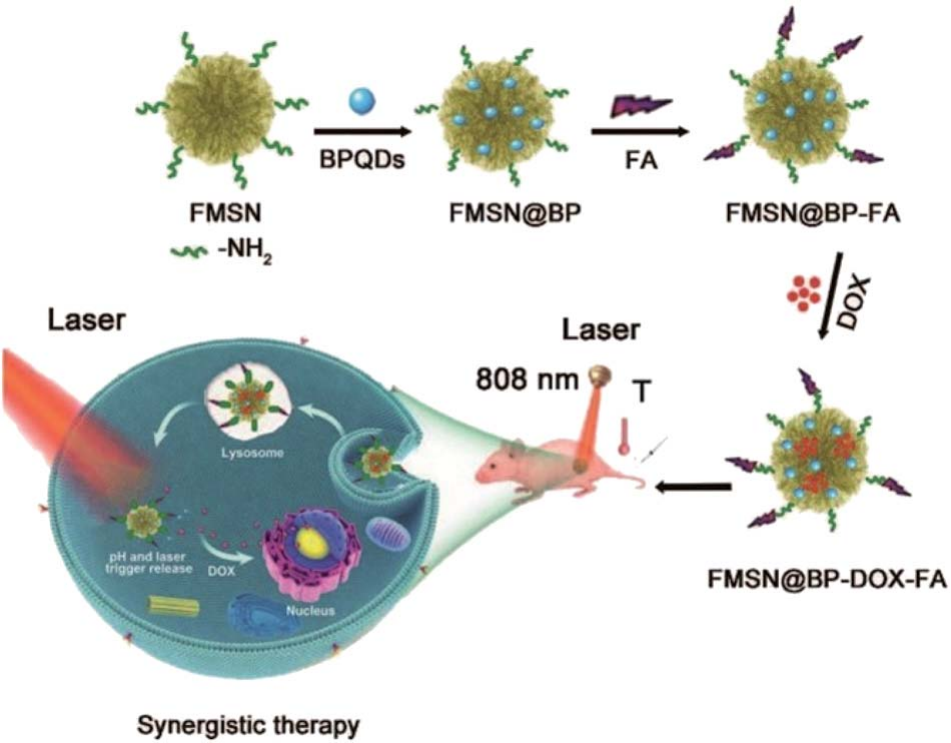
Multifunctional drug delivery system for synergistic chemotherapy and photothermal therapy.^68^ (Copyright 2020, *Nanoscale*).

Ultra-miniature BPQDs prepared by Sun *et al.* showed enhanced stability in a physiological medium and low toxicity to different types of cells. NIR photoexcitation of ultra-miniature BPQDs in the presence of C6 and MCF7 cancer cells led to significant cell death, suggesting that BPQDs had great potential as a photothermal agent for targeted photothermal cancer therapy.^67^ Ren *et al.* prepared a multifunctional drug delivery system of multifunctional layered mesoporous silica and black phosphorus nanohybrids for collaborative chemotherapy and photothermal therapy. This delivery system enhanced the loading capacity of anticancer drugs and PTT agents and improved the high photothermal conversion efficiency of PTT and the release efficiency of antineoplastic drugs such as doxorubicin (DOX) (Fig. 7).^68^

**Fig. 8:**
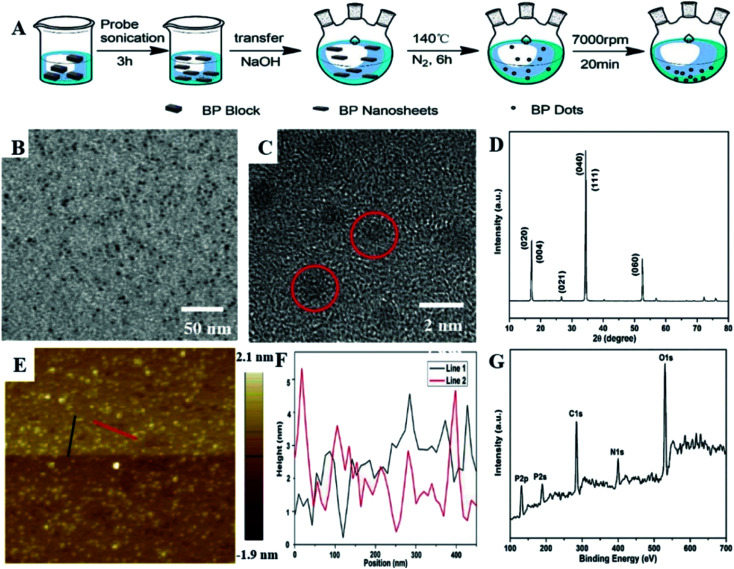
(A) Schematic diagram of the synthesis process for preparing BPQDs. (B) TEM image of BPQDs. (C) HR-TEM image of BPQDs. (D) XRD spectrum of BPQDs. (E) AFM image of BPQDs. (F) Height profiles along the red and black lines in (E). (G) XPS investigation of BPQDs.^70^ (Copyright 2018, *Analyst*).

Shao *et al.* prepared a biodegradable BPQD/PLGA (poly lactic-*co*-glycolic acid) nanosphere. Hydrophobic PLGA not only isolated internal BPQDs from oxygen and water to enhance photothermal stability, but also controlled the degradation rate of BPQDs. The results showed that BPQD/PLGA nanospheres had low toxicity and good biocompatibility. Tumor ablation under near infrared (NIR) laser irradiation showed excellent PTT efficiency and tumor targeting ability. The BPQD/PLGA nanospheres not only improved the biodegradability and biocompatibility, but also maintained high PTT efficiency, indicative of great potential for clinical applications.^69^ Wang *et al.* improved a solvothermal technique to prepare water-soluble and biocompatible BPQDs. The resulting BPQDs had a uniform size distribution and excellent biocompatibility and could effectively convert near-infrared light into thermal energy. The new nano-therapeutic agents have great potential in the photothermal therapy of cancer. BPQDs with a concentration of ≤0.5 mg mL^−1^ did not change the morphology of RBC or cause RBC cleavage. It can effectively inhibit tumor growth in tumor-bearing mice and can be excreted from the liver and kidneys (Fig. 8).^70^

**Fig. 9:**
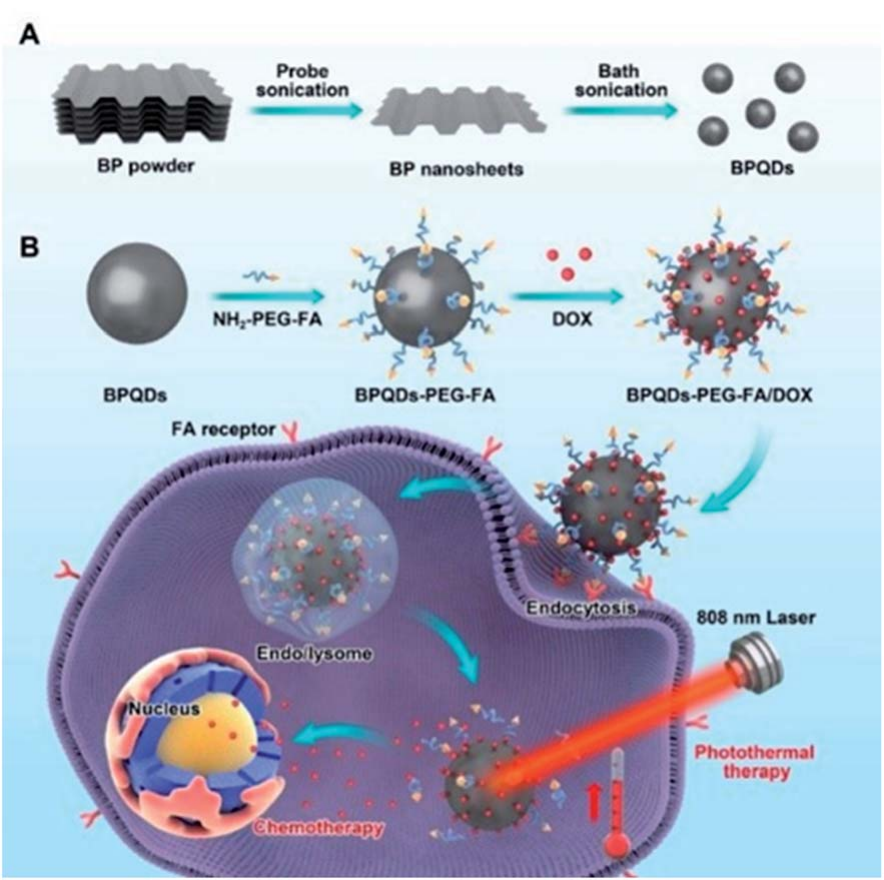
Schematic diagram of preparing black phosphorus quantum dot (BPQD)-PEG-FA/DOX. Doxorubicin and its combined chemotherapy-photothermal therapy *in vitro*. (A) Schematic diagram of the preparation of BPQDs; (B) schematic diagram of the BPQD drug delivery system. Synergistic photothermal/chemotherapy for tumors.^71^ (Copyright 2019, *Pharmaceutics*).

Luo *et al.* developed a BPQD-based drug delivery system for targeted photothermal therapy. Firstly, BPQDs were obtained by an improved liquid exfoliation method (ultrasonic treatment first and then ultrasonic bath treatment of BP powder). Then, by electrostatic action, folic acid (FA) with specific tumor targeting characteristics was bound to the surface of BPQDs and anticancer drugs were loaded for chemotherapy. The drug delivery system showed enhanced cancer cell killing ability and had great potential for clinical application in the future (Fig. 9).^71^

**Fig. 10:**
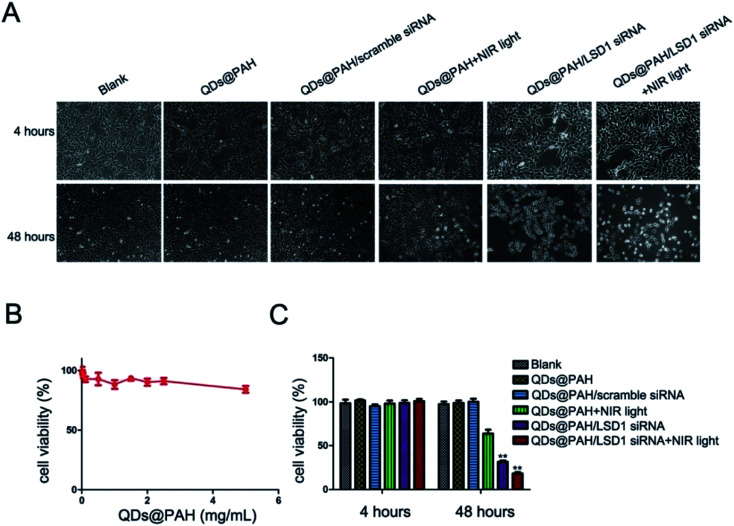
Cell viability tests of different BP-QD nanocomplexes with NIR light. The growth of PA-1 cells (cultured in a 6-well plate) was inhibited profoundly by the BP-QDs@PAH/siRNA nanocomplex with NIR light. PA-1 cells were treated with PBS, QDs@PAH, QDs@PAH/LSD1 siRNA, and QDs@PAH/scramble-siRNA for 4 hours. NIR light (808 nm continuous-wave NIR laser with a power density of 1 W cm^−2^ and a spot size of 5 mm) was utilized to treat QDs@PAH and QDs@PAH/LSD1-siRNA for 5 minutes. Then all the cells were washed with PBS and re-incubated in a fresh cell medium for designated times. Phase contrast microscopy images (A) and relative cell viabilities (C) of PA-1 cells treated with different BP-QD nanocomplexes without or with NIR light for 4 and 48 hours. (B) Viability of cells after incubation with BP-QDs@PAH at varying concentrations up to 5 mg mL^−1^ for 48 hours. Values are means ± SEM, *n* = 3; **P < 0.01 *vs.* control and QDs@PAH.^72^ (Copyright 2017, *J. Mater. Chem. B*).

Li *et al.* combined photo-thermotherapy with gene therapy for the first time. Polyelectrolyte polymers wrapped in BPQD-based nano-carriers transferred small interfering RNA (siRNA) to human ovarian teratoma PA-1 cells. The novel nano-drug BP-QDs-LSD1 siRNA inhibited the expression of lysine specific demethylase 1 (LSD1) mRNA in PA-1 cells. The transmission of LSD1 siRNA through BP-QDs@PAH showed a high internalization rate in PA-1 cells, a proliferation inhibition rate of 80%, and low cytotoxicity under NIR light (Fig. 10). This was the first application of BPQDs as a gene delivery system, which showed great potential for siRNA transmission and photothermal effects in cancer treatment.^72^

### 3.2 Photodynamic therapy (PDT)

Compared with other traditional methods in cancer therapy such as surgery, radiotherapy, and chemotherapy, PDT has become the most promising alternative therapy method. PDT relies on the combination of photosensitizers and light to cause selective damage to pathological tissues. Photosensitizers function as catalysts and produce highly reactive oxygen species (ROS), such as hydroxyl radicals (˙OH) and singlet oxygen (^1^O_2_) under appropriate light irradiation. The generated ROS can react with key cellular components, resulting in the selective destruction of tumor cells to achieve effective treatment of tumor diseases. This special mechanism grants PDT several important advantages in cancer therapy, such as little trauma, low toxicity, few side effects and good selectivity. Usually, particles smaller than 10 nm could be effectively eliminated from the kidneys and liver. BPQDs were considered as a good photosensitizer owing to their long wavelength absorption region and biodegradability.^73,74^ Li *et al.* first reported the preparation of ultra-small PEGylated BPQDs with excellent ^1^O_2_ generation capability.^75^

After that, PDT studies using BPQDs and BPQD-composites as photosensitizers have developed very rapidly. Recent studies showed that the combination of PDT and other technologies can significantly improve the therapeutic effect and reduce the side effects of treatment.^76^

**Fig. 11:**
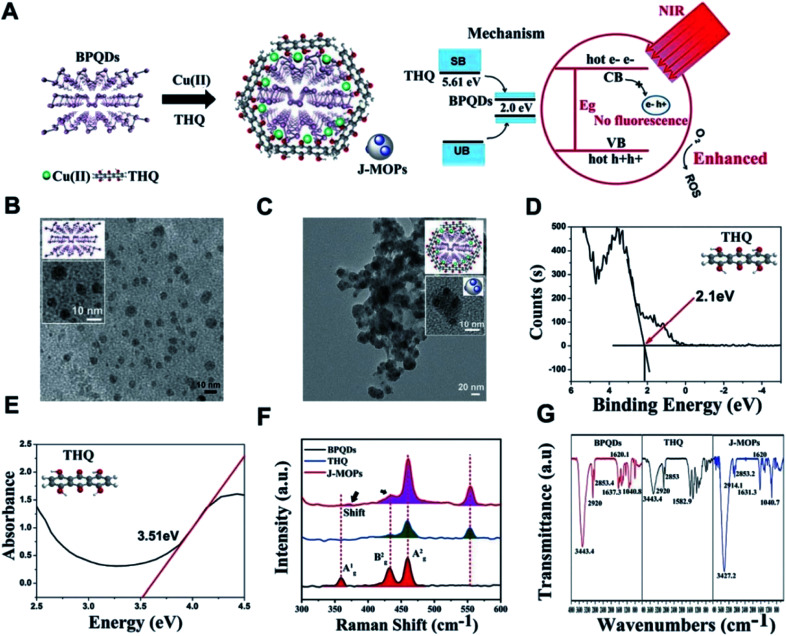
(A) Schematic illustration of the process for synthesizing J-MOPs and the mechanism of enhanced ROS generation. (B) TEM images of BPQDs and (C) J-MOPs; the inset picture and diagram present enlarged images of BPQDs and J-MOPs. (D) X-ray photoelectron spectroscopy and (E) absorbance spectra of THQ. (F) Raman spectra of BPQDs, THQ, and J-MOPs. (G) FTIR spectra of THQ, BPQDs, and J-MOPs.^77^ (Copyright 2019, *Mater. Chem. Front.*).

Zhang *et al.* designed new Janus nanoparticles (J-MOPs) based on BPQDs and THQ-Cu MOPs, which improved environmental stability and ROS formation simultaneously, and thus PDT efficiency was improved accordingly. First, the prefabricated BPQDs, THQ organic dyes and Cu^2+^ ions were mixed in aqueous solution (pH 7.4) and stirred violently for 1 h, and J-MOPs were obtained after centrifugation. The size and morphology of BPQDs and J-MOPs were characterized by transmission electron microscopy TEM (tumor microenvironment) (Fig. 11).^77^

**Fig. 12:**
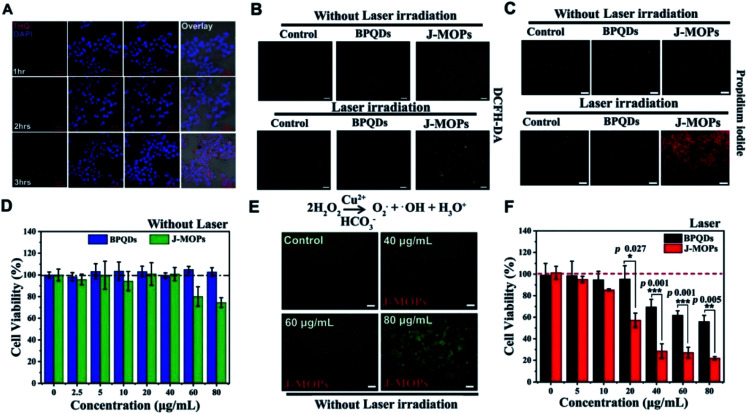
(A) CLSM images of HepG2 cells treated with J-MOPs for different incubation times. (B) DCFH-DA detection of HepG2 cells treated with PBS, BPQDs, and J-MOPs with or without 670 nm laser irradiation (0.1 W cm^−2^, 5 min; scale bars, 200 μm). (C) Corresponding fluorescence images (scale bars, 50 μm) of the treated cells stained with PI (dead cells, red fluorescence). (D) Relative cell viability of HepG2 cells after incubation with BPQDs or J-MOPs for 24 h (BPQDs, from 2.5 to 80 μg mL^−1^). (E) DCFH-DA detection of the HepG2 cells treated with J-MOPs at different concentrations (scale bars, 50 μm). (F) Cell viability of HepG2 cells after incubation with different concentrations of BPQDs or J-MOPs for 3 h, and then irradiation with a 670 nm laser (0.1 W cm^−2^) for 5 min. Statistical analysis was performed using two-tailed paired Student's *t*-tests (**P < 0.01, ***P < 0.001, and ****P < 0.0001).^77^ (Copyright 2019, *Mater. Chem. Front.*).

In this novel nanostructure, J-MOPs not only prevented BPQD degradation through effective packaging and Cu–P bonds but also endowed BQPDs with significant electron/hole separation and migration ability to enhance the production of ROS during PDT treatment. The results showed that J-MOP degradation triggered by the tumor microenvironment could release Cu^2+^ ions, which could be used as Fenton-like drugs, and further improved the anti-tumor efficacy by synergistic action with PDT. Therefore, they emphasized the potential of the designed J-MOPs as a promising phototherapy agent for cancer in possible clinical applications (Fig. 12).^77^

**Fig. 13:**
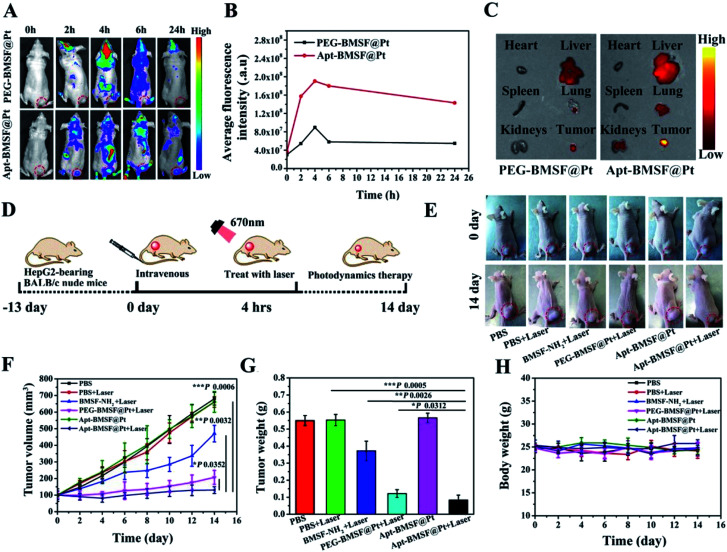
(A) *In vivo* fluorescence imaging of PEG-BMSF@Pt and Apt-BMSF@Pt at different time points after intravenous injection. (B) Quantification of the fluorescence intensity of tumors after intravenous injection at different time points. (C) *Ex vivo* fluorescence images of the tumor and organs isolated from HepG2-tumor bearing mice after 4 h of Cy5 labeled PEG-BMSF@Pt or Apt-BMSF@Pt treatment, respectively. (D) Schematic illustration of a typical therapeutic procedure. (E) *In vivo* response to PBS, BMSF-NH_2_, PEG-BMSF@Pt and Apt-BMSF@Pt with or without laser irradiation; (F) tumor volume changes after indicated treatments (*n* = 5). (G) The average tumor weight of mice after indicated treatments (*n* = 5); (H) mean body weight of mice after indicated treatments (*n* = 5).^78^ (Copyright 2019, *ACS Appl. Mater. Mater Interfaces*).

Zeng *et al.* designed an (Hepatocellular carcinoma HCC) HCC-specific aptamer TLS11a grafting nano-catalyst based on a BPQDs/hybridized mesoporous silica framework. The results showed that the nano-system modified with TLS11a aptamer/Mal-PEG-NHS (called Apt-BMSF@Pt) had excellent environmental stability and active targeting ability towards HCC cells, as well as oxygen self-compensation ability. The experimental results showed that Apt-BMSF@Pt could effectively accumulate at the tumor site, and the core of BMSF acted as a photosensitizer to generate ROS, while PtNP could act as a catalyst to convert H_2_O_2_ into O_2_ to enhance the effect of PDT. By comparing the tumor volume/weight, type and immunohistochemical results, it was proved that Apt-BMSF@Pt had an excellent anti-tumor effect and minimal side effects (Fig. 13).^78^

**Fig. 14:**
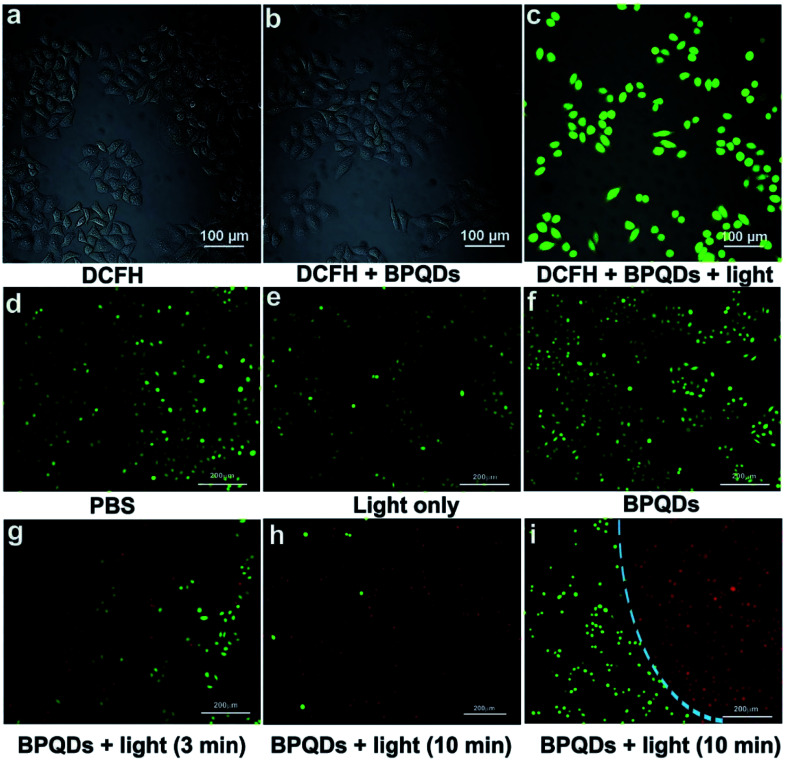
Merged confocal fluorescence microscope images of HeLa cells after different treatments: (a) incubated with DCFH-DA, (b) incubated with DCFH-DA and BPQDs, and (c) incubated with DCFH-DA and BPQDs under light irradiation. Merged inverted fluorescence microscope images of HeLa cells after different treatments: (d) incubated with PBS, (e) light irradiation only, (f) incubated with BPQDs only, (g and h) incubated with BPQDs (1.6 μg mL^−1^) and then treated with light irradiation (670 nm, 160 mW cm^−2^) for 3 and 10 min, respectively, and (i) treated with BPQDs (1.6 μg mL^−1^) and light irradiation (670 nm, 160 mW cm^−2^, and 10 min); the cells partly covered with the light spot showing the boundary. The cells are all constrained by calcein-AM (green, live cells) and propidium iodide (red, dead cells).^79^ (Copyright 2018, *Small*).

**Fig. 15:**
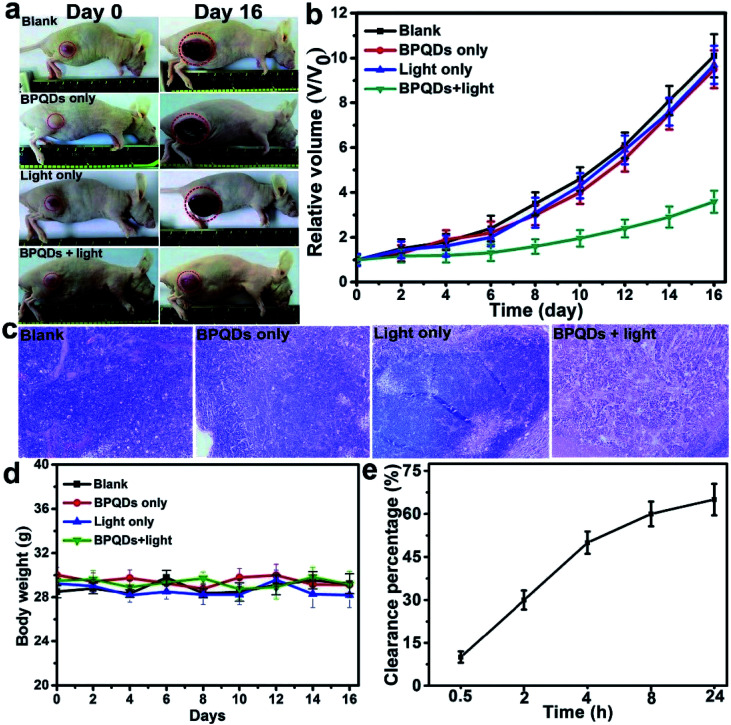
(a) Representative photographs of S180 tumor-bearing nude mice before and after different treatments. Photos were collected on day 0 and day 16, respectively. (b) Tumor growth curves, (c) H&E-stained histological images of tumor sections, and (d) body weight changes of the S180 tumor bearing mice after different treatments. (e) Cumulative clearance percentage of BPQDs determined by measuring the P content in urine at different time points after the administration (*n* = 5).^79^ (Copyright 2018, *Small*).

The BPQDs prepared by Huang-Hao Yang *et al.* can be quickly cleared from the body in their complete form through the kidneys. *In vitro* and *in vivo* studies showed that BPQDs had an excellent photodynamic effect under light irradiation and could effectively produce ROS to kill cancer cells (Fig. 14 and 15).^79^

### 3.3 Drug delivery

Similar to 2D BP nanosheets, BPQDs also showed high drug loading capacity attributed to the large surface-area-to-volume ratio. Combined with great biocompatibility and degradability, BPQD and BPQD complexes have become important drug delivery platforms in recent studies.^80^

**Fig. 16:**
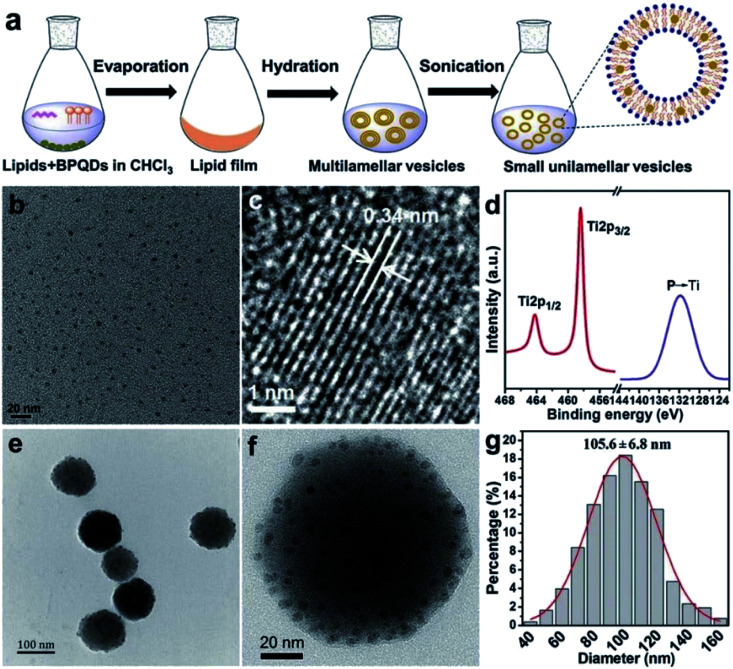
(a) Preparation of BPQDs@Lipo; (b) TEM image of BPQDs; (c) HR-TEM image of BPQDs; (d) HR-XPS spectra of P 2p and Ti 2p; (e) TEM image of BPQDs@Lipo; (f) HR-TEM image of BPQDs@Lipo; and (g) size distribution of BPQDs@Lipo measured by DLS.^81^ (Copyright 2018, *Chem. Commun.*).

**Fig. 17:**
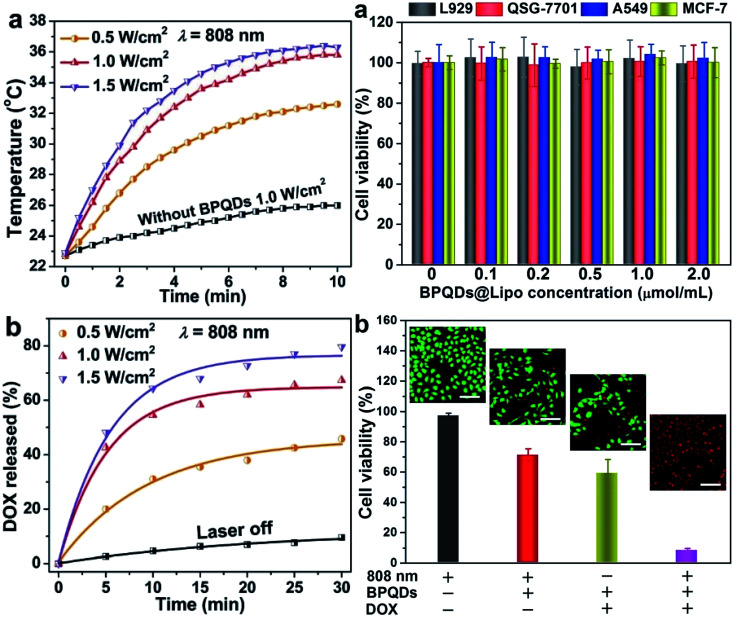
(a) Photothermal heating curves of bare liposomes and BPQDs@Lipo under 808 nm laser irradiation at different power densities; (b) DOX release profiles of BPQDs@Lipo under 808 nm laser irradiation at different power densities.^73^ (Copyright 2018, *Chem. Commun.*). The cytotoxicity of BPQDs@Lipo was evaluated by using two normal cell lines L929 (mouse fibroblasts) and QSG-7701 (normal hepatocytes) and two cancer cell lines A549 (human). The results show that the BP-liposome complex has great clinical potential in multimodal cancer therapy.^81^ (a) Relative viabilities of L929, QSG-7701, A549, and MCF-7 cells after incubation with different concentrations of BPQDs@Lipo for 24 h assessed using a CCK-8 kit (*n* = 6). (b) Relative viabilities of the MCF-7 cells for different treatments: NIR light irradiation (an 808 nm laser of 1.0 W cm^−2^ for 10 min), BPQDs@Lipo with NIR light irradiation, BPQDs@LipoDOX, and BPQDs@Lipo-DOX with NIR light irradiation. The cell viability is evaluated using a CCK-8 kit (*n* = 6) and the scale bar is 100 mm.^81^ (Copyright 2018 *Chem. Commun.*).

Geng *et al.* prepared a BPQD liposome complex (BPQDs@Lipo) for a NIR optically controlled drug delivery system by doping BP into liposome bilayers. The embedding of biocompatible materials in the bilayer in response to some stimuli can be controlled by light intensity and exposure time (Fig. 16). Due to the photothermal effect of BPQDs, the encapsulated drug will be released from the liposome under 808 nm laser irradiation (Fig. 17).^81^

**Fig. 18:**
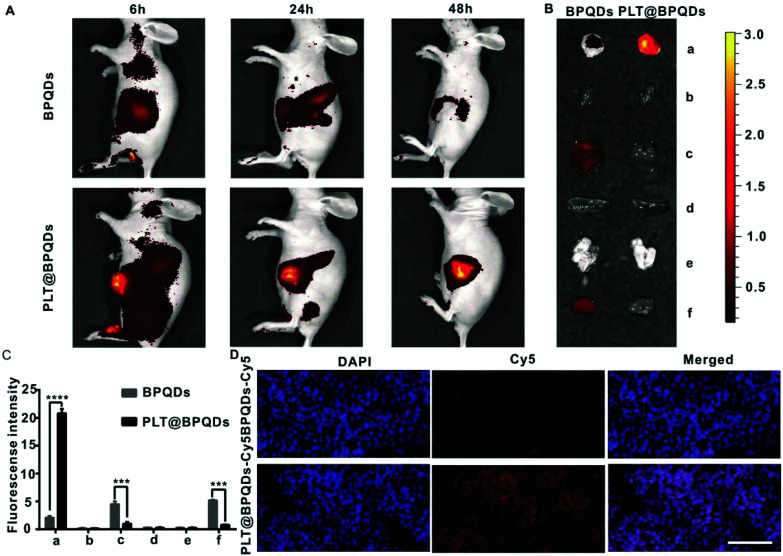
*In vivo* targeting potential of PLT@BPQDs-HED. (A) *In vivo* fluorescence images of nude mice at 6 h, 24 h and 48 h upon intravenous treatment with Cy5-linked BPQDs and Cy5-labeled PLT@BPQDs. (B) *Ex vivo* bioluminescence images of visceral organs and tumors at 48 h post-treatment with Cy5-conjugated BPQDs and Cy5-linked PLT@BPQDs. (a) Tumor, (b) heart, (c) liver, (d) spleen, (e) lung, and (f) kidney are shown. (C) Semiquantitative assessment of the fluorescence signals of the tumor and other tissue specimens. (a) Tumor, (b) heart, (c) liver, (d) spleen, (e) lung, and (f) kidney are shown. (D) Fluorescence imaging of tumor tissues from nude mice after 48 h upon the administration of Cy5-linked BPQDs and Cy5-conjugated PLT@BPQDs. Scale bar: 100 μM. Data are mean ± SD (*n* = 3). Compared to the BPQD group: *p < 0.05, **p < 0.01, ***p < 0.001, and ****p < 0.0001.^82^ (Copyright 2019, *ACS Appl. Mater. Interfaces*).

Gui *et al.* prepared a BPQDs-hederagenin (HED) nanocomposite PLT@BPQDs-HED encapsulated by platelet membrane (PLTm) vesicles targeting tumor cells. It could enhance the cancer therapeutic effect and reduce the side effects of free HED. BPQDs showed a high drug loading rate and excellent safety. Under acidic conditions, the accelerated degradation of BPQDs promoted the release of HED from PLT@BPQDs-HED in the tumor acidic microenvironment, and the immune system cannot remove the camouflaged BPQDs, so it could remain in the tumor for a long time. Platelets (PLTs) were bound to tumor cells through P-selectin and the CD44 receptor. It was a non-toxic and effective targeted drug delivery platform for tumor therapy. The platform could enhance the anti-tumor activity of HED by regulating apoptosis and autophagy, while reducing the side effects of myelosuppression. PLT@BPQDs-HED nanocomposites showed a variety of anti-tumor mechanisms in cancer therapy in that study, indicating their high clinical application value (Fig. 18).^82^

### 3.4 Biological imaging

In addition to PTT, PDT and drug deliver applications, BPQDs were also demonstrated to be biocompatible fluorescence agents. Compared with conventional metal-based semiconducting quantum dots with long-term toxicity and environmental problems, BPQDs had excellent biocompatibility and low toxicity in human systems. These properties are important for BPQDs to be considered as an ideal candidate material for biological imaging and biomarkers.^83^

**Fig. 19:**
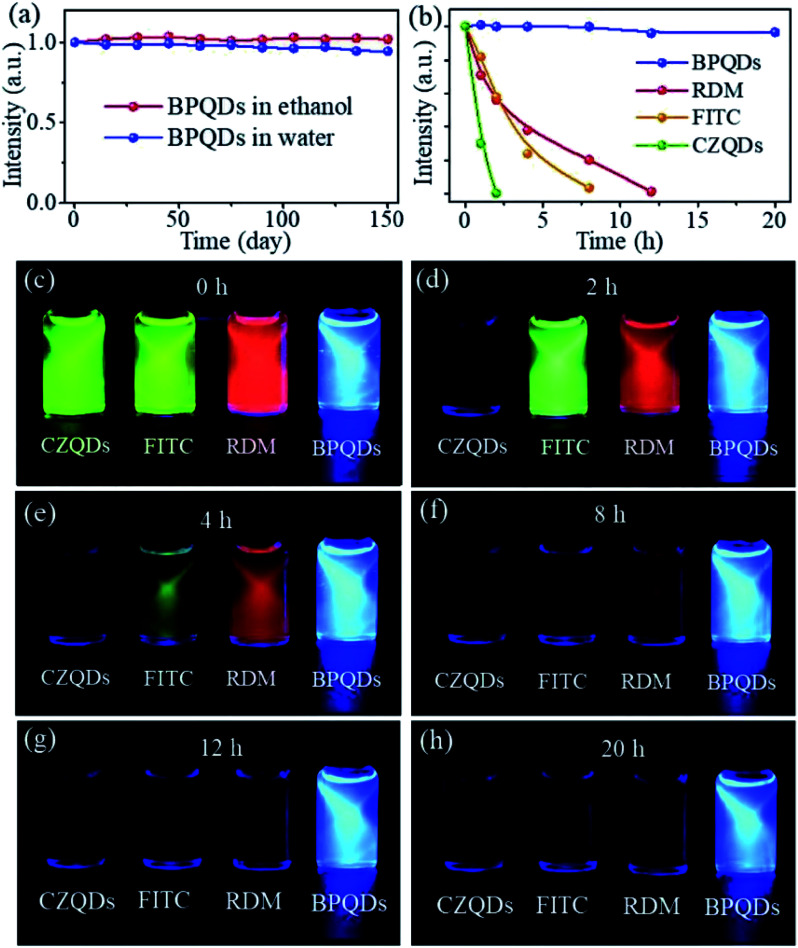
(a) Emission intensity of P-BPQDs in ethanol and water *versus* storage time in an ambient environment for a total storage time of 150 days. Each PL measurement is performed with 100 W Xe lamp illumination. (b) Peak intensity of P-BPQDs in water, RDM, FITC, and CZQDs *versus* illumination time (Xe lamp). The PL measurement conditions and parameters are the same and intensities are normalized according to the initial intensity value. (c–h) Luminescence photographs of P-BPQDs in water and three contrast samples (CZQDs, FITC, and RDM) after continuous irradiation with a Xe lamp for different times. The photographs are collected using the same parameters with a camera.^84^ (Copyright 2018, *Small*).

**Fig. 20:**
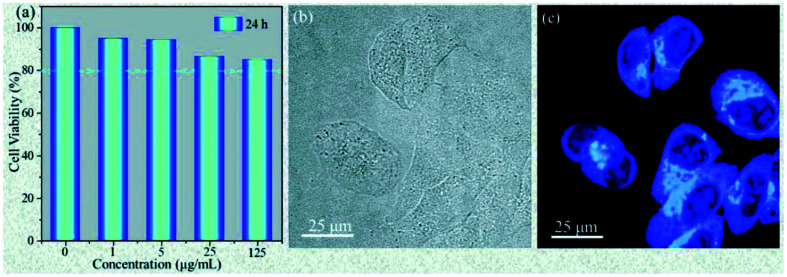
Cellular toxicity and bioimaging tests of P-BPQDs. (a) Cell viability of the HeLa cells after incubation for 24 h with P-BPQDS solutions of different concentrations. (b and c) Confocal microscopy images of the HeLa cells labeled with the P-BPQDs under a bright field and dark field under the excitation of a 405 nm laser line.^84^ (Copyright 2018, *Small*).

Wu *et al.* prepared highly fluorescent and stable BPQDs by a liquid stripping method using ultrasonic probe treatment and solvent heat treatment in ethanol. Compared with previously reported results, BPQDs obtained by this new method exhibited an excellent photoluminescence (PL) quantum yield, as high as 70% in water. And the PL stability for 150 days in the surrounding environment was also very high. The experimental results showed that the photostability of the prepared BPQDs was better than that of many heavy metal semiconductor nanostructures and organic fluorescent dyes with high PL, mainly resulting from the quantum size effect and the introduction of two surface states related to P–OH and P–O–CH_2_CH_3_ by the ethanol treatment (Fig. 19). Polar water molecules removed non-radiative centers and increased P-related fluorescent groups, which enhanced PL by effective carrier transfer to the surface radiation state. Taking advantage of the excellent fluorescence characteristics, BPQDs had excellent performance in cell labeling with good biocompatibility, showing great potential for biological imaging (Fig. 20).^84^

**Fig. 21:**
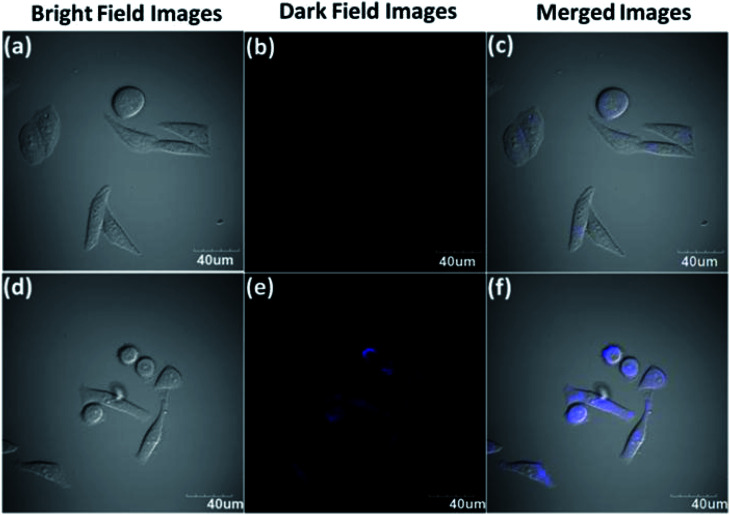
(a)–(c) Confocal microscopy images of live HeLa cells without the treatment of BPQDs. (d)–(f) Confocal microscopy images of live HeLa cells after 3 h incubation with 50 μL BPQDs at 37 °C. Detection of BPQD fluorescence was achieved under excitation at 405 nm.^85^ (Copyright 2018, *Chem.–Asian J*.).

Zhang *et al.* successfully synthesized several layers of BPQDs through a pulsed laser ablation (PLA) method in isopropyl ether (IPE) solvent. The prepared sample had a wrinkled hexagonal structure, which showed stable blue-purple luminescence and a relatively high PL quantum yield (about 20.7%) for the first time after 15 days, which was 3 times higher than that of BPQDs prepared by ultrasonic exfoliation using a probe (about 7.2%). The prepared BPQDs were used for biological imaging of HeLa cells, showing a good PL signal and excellent biocompatibility. This study showed that BPQDs with a high quantum yield and stable PL emission can be used in medical biological imaging research (Fig. 21).^85^

### 3.5 Other biomedical applications

#### 3.5.1 Interaction with human serum albumin (HSA)

**Fig. 22:**
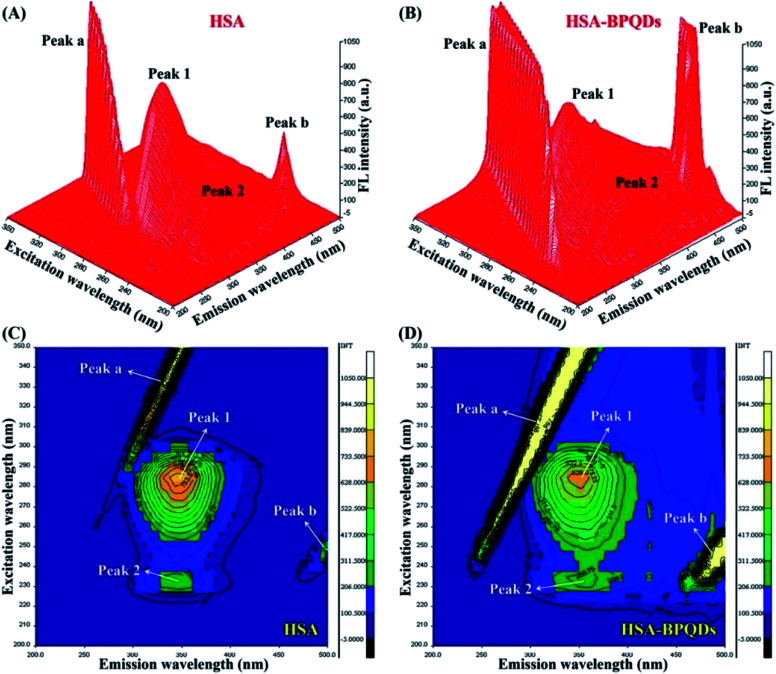
Three-dimensional fluorescence spectra of HSA (A) and HSA-BPQD systems (B). Contour maps of the three-dimensional fluorescence spectra of HSA (C) and HSA-BPQD systems (D). The concentrations of HSA and BPQDs were 2.0 × 10^−6^ and 2.0 × 10^−5^ mol L^−1^, respectively.^86^ (Copyright 2020, *Int. J. Biol. Macromol.*).

Liu *et al.* found that BPQDs could affect the conformational structure variation of HSA. The intrinsic fluorescence of HSA is statically quenched by BPQDs mainly through van der Waals interaction and hydrogen bonding, and a ground state complex with a molar ratio of 1 : 1 is formed. When combined with BPQDs, the transformation from an α-helix structure into a β-sheet structure of the secondary structure of HSA results in the decrease of thermal stability of HAS (Fig. 22).^86^

#### 3.5.2 Inhibit protein aggregation

**Fig. 23:**
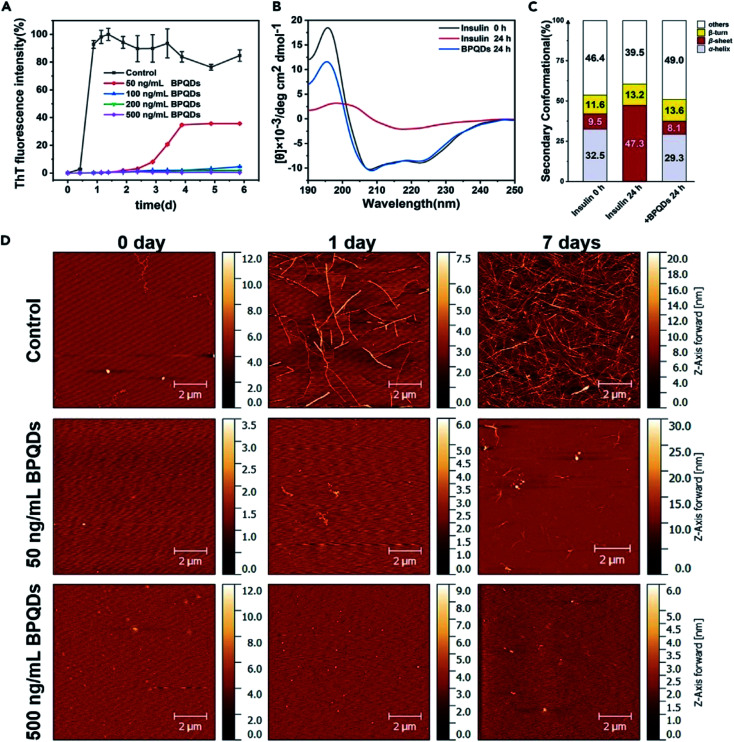
(A) The growth kinetic curves of insulin amyloid fibrillation monitored by ThT fluorescence assay. Relative ThT fluorescence intensity varied at 485 nm with the incubation time of insulin at different BPQD concentrations (0, 50, 100, 200, and 500 ng mL^−1^). The final concentration of insulin is 2 mg mL^−1^. All data represent the average from three experiments. Error bars indicate GSD. (B) Far-UV CD spectral curves of insulin alone and in the presence of 500 ng mL^−1^ of BPQDs, as well as the corresponding secondary structure content. (C) The primal insulin and the incubated (24 h) insulin were diluted twice and tested using a 1 nm quartz cell. (D) AFM images of insulin amyloid fibrillation at different incubation times (0, 1, and 7 days) for insulin alone and insulin BPQD (50 and 500 ng mL^−1^) solutions. (AFM: tapping mode).^87^ (Copyright 2020, *iScience*).

**Fig. 24:**
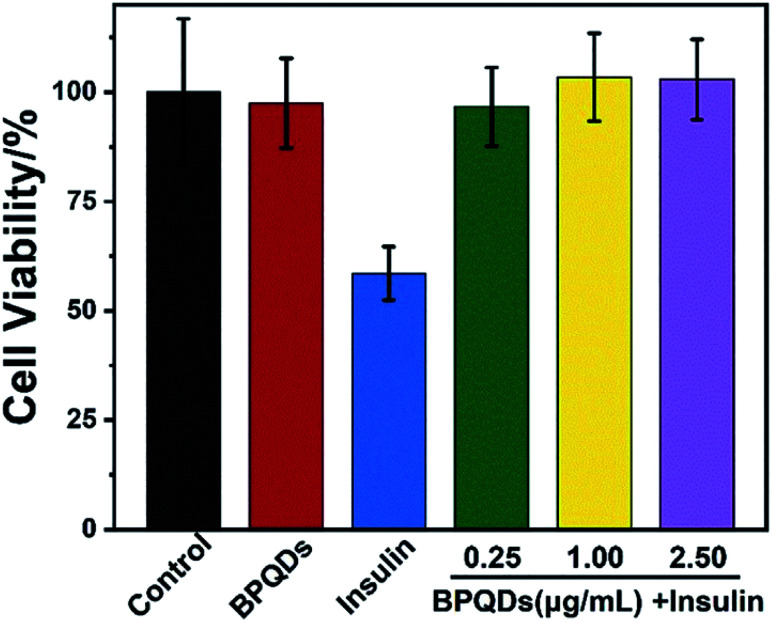
Cell viability was measured by the MTT reduction method. PC12 cells were treated with BPQDs alone, insulin alone, and insulin in the presence of BPQDs after 5 days of incubation. The concentrations of BPQDs were 0.25, 1.00, and 2.50 mg mL^−1^, respectively. The final concentration of insulin was 5 mM. The control experiment was performed without insulin fibrils. All data represent the average from six different data. Error bars indicate GSD.^87^ (Copyright 2020, *iScience*).

Su *et al.* chose insulin as a model protein and found that the addition of BPQDs significantly inhibited the transformation of insulin into amyloid fibrils at a very low concentration within a few days. The cytotoxicity of BPQD and insulin solutions exposed to different concentrations of BPQDs was evaluated, and the results showed that BPQDs at an ultra-low concentration can be used as an effective and non-toxic inhibitor of amyloid fibers, which provides a new idea for the treatment of diabetes and other diseases involving amyloid fibrillation (Fig. 23 and 24).^87^

#### 3.5.3 Nephrotoxicity

Ma *et al.* used a kidney-like organ model to simulate the function of the kidneys in the body. Using the renal organ model, BPQDs were screened and the adverse effects of BPQD accumulation in the kidneys were evaluated. The nephrotoxicity of BPQDs can be further verified by using both mouse and human cells, and it has been proved for the first time that BPQDs cause insulin insensitivity and endoplasmic reticulum stress in the kidneys. It is further clarified that IRE1 α signal transduction associated with endoplasmic reticulum stress mediates nephrotoxicity and insulin sensitivity induced by BPQDs.^88^

## 4. Summary and future prospects

Since the discovery of BPQDs, researchers have been trying to find different ways to synthesize BPQDs. At present, liquid phase stripping technology still remains the most widely used method in the synthesis of BPQDs. Other synthetic methods mainly include electrochemical stripping, solvent heat treatment, agitator crushing and pulsed laser irradiation. The synthetic methods of BPQDs have been thoroughly discussed in previous review papers and no major breakthroughs have been achieved in the last two years, and thus the synthetic methods of BPQDs will not be discussed in detail in this work. Table 1 summarizes the synthesis methods and main applications of BPQD based nanomaterials reported since 2018.

**Table 1:** Summary of BPQD based nanomaterials prepared since 2018, including synthetic methods, fields of applications, and major innovations

Therapeutic platform	Synthetic methods	Application area	Highlight	Ref.
BPQDs	Liquid stripping technology	Cancer treatment	Ultra-small size	26
			Excellent NIR photothermal performance	
FMSN@BP	Electrochemical stripping	Cancer treatment	Improvement in the efficiency of PTT photothermal conversion and the release efficiency of anti-tumor drugs (such as DOX)	68
BPQDs/PLGA NSs	Liquid stripping technology	Cancer treatment	Efficient photothermal cancer treatment	69
BPQDs-PEG-FA	Liquid stripping technology	Cancer treatment	Targeted chemotherapy and photothermal therapy	71
BP-QDs@PAH	Liquid stripping technology	Cancer treatment (especially CSC treatment)	High transfection efficiency, biocompatibility and non-toxicity	72
J-MOP		Antitumor	Enhance ROS production	77
Apt-BMSF@Pt	Liquid stripping technology (ultrasonic peeling technology)	Hepatocellular carcinoma (HCC)	Prevention of BPQD oxidation. Specific targeting in HCC cells. Enhancement of PDT	78
BPQDs	Liquid stripping technology (ultrasonic probe peeling technology)	Bioimaging of HeLa cells	High quantum yield. Stable PL launch	85
BPQDs/Ti_3_C_2_@TiO_2_	Liquid exfoliation technology	Photocatalytic activity	Enhanced photocatalytic activity	90
BPQDs	Liquid stripping combined with an ultrasonic probe technique	Cancer treatment	Synergistic enhancement of mild PTT therapy	91
PEGylated BPQDs	Liquid stripping technology	Cancer bioimaging. Combination of photothermal therapy (PTT) and photodynamic therapy (PDT)	Excellent photodynamic properties. The combination of PTT and PDT phototherapy improves the efficacy of cancer	92
RdB/PEGBPQDs				
PLGA-SS-D@BPQDs	Emulsion evaporation	Radiosensitizer. Cancer targeted therapy	Efficient radiation therapy. Low toxicity and few side effects	93
BPQD-RMNVs	Liquid stripping technology	Breast cancer	Induction of triple negative breast cancer cell apoptosis	94

Compared with traditional 2D BP nanosheets, BPQDs possess higher band gaps, smaller sizes, higher surface-to-volume ratios and more active edge sites, which results in many unique and fascinating properties of BPQDs. With the advantages of strong light absorption, high photothermal conversion efficiency and good biocompatibility, BPQDs have broad research and application prospects in the biomedical field, such as biological imaging, drug delivery, photothermal therapy, photodynamic therapy and so on. Compared with the commonly used materials in the biomedical field (including metal nanoparticles, carbon-based nanomaterials, and other 2D materials such as titanium disulfide and molybdenum disulfide), BPQDs are made of phosphorus and can be degraded into phosphoric acid in the human body, which is harmless. *In vivo* safety is a key issue for the successful clinical applications of BPQD based nanomaterials.

In this burgeoning new field, many new challenges still remain, and many topics still need to be studied in depth. The preparation of BPQDs is still limited to the laboratory scale, which is far from sufficient to meet the requirements of future practical applications. It is urgent to develop a robust and effective method to prepare BPQDs in high-quality and large quantity. Although phosphorus is a friendly element in the human body, the *in vivo* safety of BPQDs still needs systematical long-term investigations. The degradation mechanisms of BPQD and BPQD composites in the human body still need to be further studied. In recent years, continuous progress has been achieved in solving the problems of extreme unitability of BPQDs in water and poor dispersion of BPQDs in aqueous solutions. Despite that, the problems of stability and dispersion are still the focus of future research. The applications of BPQD based nanomaterials as photosensitizers in PDT therapy and radiosensitizers in radiation therapy have been reported, but the working mechanisms and structure–activity relationships of BPQD based nanomaterials are still under investigation. In recent studies, composite materials and hybrid systems for functional modifications of BPQDs have been widely used in cancer treatment, which exhibit improved therapeutic effects with low toxicity and high biocompatibility. These BPQD based composites and hybrid systems can overcome the disadvantages of BPQDs and have shown excellent applications in many aspects, such as biological imaging and drug delivery. This is an important direction for further research on BPQD based nanomaterials.

In summary, this review summarizes the recent advances in BPQD based biomedical applications, especially those reported in the last two years. BPQD and BPQD based nanomaterials have been successfully applied in various biomedical applications including PTT, PDT, drug delivery and biological imaging, among others. Despite impressive progress and rapid development in this field, BPQDs still face several unsolved challenges, such as preparation methods, stability and dispersion in aqueous solutions. The long-term *in vivo* safety and working mechanisms of BPQD based nanomaterials also need further investigations.

## Conflicts of interest

The authors declare no competing financial interest.

## Supplementary Material
